# Notes from the Field: *Cronobacter sakazakii* Infection Associated with Feeding Extrinsically Contaminated Expressed Human Milk to a Premature Infant — Pennsylvania, 2016

**DOI:** 10.15585/mmwr.mm6628a5

**Published:** 2017-07-21

**Authors:** Anna Bowen, Harold C. Wiesenfeld, Jennifer L. Kloesz, A. William Pasculle, Andrew J. Nowalk, LuAnn Brink, Elisa Elliot, Haley Martin, Cheryl L. Tarr

**Affiliations:** ^1^Division of Foodborne, Waterborne and Environmental Diseases, National Center for Emerging and Zoonotic Infectious Diseases, CDC; ^2^University of Pittsburgh, Pennsylvania; ^3^Magee-Womens Hospital of UPMC, Pittsburgh, Pennsylvania; ^4^UPMC Presbyterian Clinical Microbiology Laboratory, Pittsburgh, Pennsylvania; ^5^Children's Hospital of Pittsburgh of UPMC, Pennsylvania; ^6^Allegheny County Health Department, Pennsylvania; ^7^Coordinated Outbreak Response and Evaluation Network, Food and Drug Administration, Silver Spring, Maryland.

In April 2016, a female infant was born via Cesarean delivery at 29 estimated gestational weeks and had a birthweight of 3 pounds (1,405 grams). Her clinical course in the neonatal intensive care unit was unremarkable until she developed signs of sepsis at age 21 days. Cultures of blood and cerebrospinal fluid yielded *Cronobacter sakazakii*, a gram-negative pathogenic bacillus. Despite treatment with ampicillin and cefepime, she developed seizures; brain imaging revealed liquefaction necrosis of the entire left cerebral hemisphere and right frontal lobe. The infant developed spastic cerebral palsy and global developmental delay and required a ventriculoperitoneal shunt and a gastrostomy feeding tube.

The infant had been fed pasteurized donor human milk and expressed maternal milk (EMM) during the first week after birth; thereafter, she received EMM mixed with a commercial liquid human milk fortifier. Maternal milk was expressed using a dedicated bedside hospital breast pump and the mother’s personal breast pump throughout the infant’s hospitalization. The infant did not receive powdered infant formula products.

Clinicians and microbiologists from the hospital, Allegheny County Health Department, the Food and Drug Administration, and CDC investigated the source of the infection. Items and materials tested included the personal and hospital breast pump kits; samples of frozen EMM from the personal breast pump; hand-expressed maternal milk; lanolin used to treat the mother’s breasts; human milk fortifier, caffeine citrate, vitamin D, and iron supplements from lots given to the infant; tap water, faucet and sink surfaces from the hospital bedside and home kitchen; two wash basins from the home kitchen; and maternal stool samples. *C. sakazakii* was cultured from the valves of the personal breast pump kit, 11 frozen EMM samples collected using that pump kit during 7 separate days before illness onset, and the drain of the kitchen sink at the mother’s home. Cultures of the personal breast pump kit and the 11 frozen EMM samples each yielded 2–5 additional gram-negative bacteria; other items did not yield pathogens. Except for the EMM isolate with the earliest collection date, pulsed-field gel electrophoresis patterns of all EMM and clinical *C. sakazakii* isolates were indistinguishable.

The mother reported typically soaking the collection kit from her personal breast pump in soapy water in a wash basin for ≤5 hours without scrubbing or sanitizing. She then rinsed, air-dried, and stored the kit in a plastic zip-top bag until the next use. The collection kit from the hospital breast pump was washed immediately and thoroughly air-dried at the bedside. The mother did not report symptoms or signs of mastitis.

*C. sakazakii* can cause sepsis and severe meningitis, particularly among infants (*1*). *Cronobacter* infections have been traced to contaminated powdered infant formula, and only once has a source unrelated to powdered infant formula been reported to be associated with an infant infection (*1*,*2*). However, *Cronobacter* can be found in other food products and the environment, and some infected infants did not consume powdered infant formula (*1*,*3*). This case of *C. sakazakii* infection caused by consumption of extrinsically contaminated expressed human milk led to meningitis, brain necrosis, and marked developmental delays. Human milk is the ideal food for nearly all infants and is associated with decreased risk for many illnesses; however, microorganisms can multiply rapidly in expressed human milk (*4*). Although many women report good hygiene while expressing milk (*5*), expressed human milk is frequently contaminated with pathogens (*6*,*7*), most likely because of suboptimal hygiene practices associated with milk expression. Although the source of contamination in this case is unknown, a breast pump kit became contaminated with *C. sakazakii* and was not adequately cleaned or sanitized, leading to contamination of the milk expressed with this kit on several days. Human milk contaminated during or after expression can put infants at risk for infection with various pathogens, including *C. sakazakii* (*7*). CDC has developed guidance to optimize breast pump hygiene.* Clinicians should provide detailed recommendations about hygienic expression and handling of human milk to parents who plan to feed EMM to their infants. Settings in which mothers might need to pump their milk, such as hospitals and workplaces, should facilitate hygienic expression and handling of human milk.

## Footnotes

* https://www.cdc.gov/healthywater/hygiene/healthychildcare/infantfeeding/breastpump.html.
